# Predictors of maternal parenting self-efficacy for infants and toddlers: A Jordanian study

**DOI:** 10.1371/journal.pone.0241585

**Published:** 2020-11-18

**Authors:** Sawsan Abuhammad

**Affiliations:** Faculty of Nursing, Jordan University of Science and Technology, Irbid, Jordan; University of Iowa, UNITED STATES

## Abstract

**Aim:**

This study aimed to investigate the predictors of maternal parenting self-efficacy when the children concerned are in the early years of life.

**Method:**

A descriptive-analytical research study was carried out among 213 women who were in the early months of the postpartum period and attending healthcare facilities in Irbid, Jordan. The State Anxiety Inventory (SAI) and the Maternal Parenting Self-Efficacy (PMP S-E) tool were used to collect the data.

**Results:**

A significant correlation was found between the scores in self-efficacy and the quality of marriage relations (B = 3.56, P = 001), family income (B = 1.97, P = .05), employment (B = 4.027, P = .027), education (B = 2.48, P = .004), and living with extended family (B = 5.28, P = .02).

**Conclusion:**

The findings of this study show that MPSE is significantly associated with various predictors. These predictors are the mother’s education, income, whether she lives with extended family, her quality of marriage, and her employment. Maternal anxiety was found not to be a predictor for MPSE and this may explain other factors such as social support and living with extended family.

**Implication:**

It is essential for nurses to understand maternal parenting self-efficacy, therefore, including the concept of maternal parenting self-efficacy in nursing curricula can help raise awareness of this important concept. Understanding maternal parenting self-efficacy is necessary for nurses to evaluate the mothers’ parenting self-efficacy.

## Introduction

Maternal parenting self-efficacy (MPSE) is one of the critical elements of maternal transformation as a woman moves into parenthood. A mother’s self-efficacy as a parent not only affects her own mental health, it also influences the psychological development of her infant [1, 2].The postpartum period can be a depressing stage in any parent’s life, but particularly in women because it is during this time that the reality of caring for a newborn becomes tangible [3]. If a woman does not get Information on infant and toddler cues/behaviors so that mothers can develop sensitivity and responsiveness toward their infants during this time, she may not only experience low MPSE, her health and that of her infant and toddler may also be adversely affected [4, 5].

Thus, to enable a seamless transition into parenting, it is essential to be aware of the factors that affect MPSE in order to facilitate the planning and delivery of comprehensive care to disadvantaged mothers. According to Bandura, MPSE refers to the perceptions that mothers have regarding their ability to accomplish particular duties in caring for newborns [6]. Mothers with high MPSE levels have high persistency when they encounter problems or challenges in caring for infants and have a high likelihood of initiating beneficial action plans and flexible adjustments [6, 7]. Therefore, Bandura asserts that MPSE levels dictate the inception of maternal attempts and the steady accomplishment of duties in newborn care in the early years of a child’s life [6].

According to Yap, Nasir, Tan, and Lau, the level of MPSE is dependent on various factors, such as individual assets, society, and the environmental context [8]. The factors that play a critical roles in promoting MPSE are (a) maternal characteristics, for example, age, socio-economic status, childcare experience, and emotional state, (b) characteristics of the child, for example, health status, impatience, and attitude, and (c) attributes of the environment, for example, the functioning of the family, contentment in marriage, and social and cultural upbringing [9, 10].

A review of eight quantitative studies on MPSE concluded that MPSE has a positive relationship with multiparty support, the support of society, parenting, and contentment in marriage, while it has a negative association with maternal stress and state of anxiety [10]. Leahy-Warren, McCarthy, and Corcoran, who investigated the self-efficacy of American couples before child delivery and at four months postpartum, found that social support had an association with the self-efficacy of the mothers and found that anxiety impacted MPSE negatively [11]. In addition, a study by Salonen et al. on the self-efficacy of 1,300 families in Finland revealed that multiparty support, aspects of social assistance, and symptoms of depression have a significant impact on the mother’s self-efficacy [12]. This means multiparity support, social assistance, and depression all impact MPSE in the same way. Higher support leads to higher SE and worse depression lower or higher MPSE. Moreover, a study that investigated self-efficacy in Singaporean women highlighted that stress and maternal contentment in caring for newborns were key predictors of self-efficacy [13]. Furthermore, a report by Gao, Sun, and Chan indicates that among 68 women in China, social assistance strongly predicts self-efficacy. Previous studies have utilized different designs and instruments to investigate maternal self-efficacy, which makes it hard to perform comparisons [14]. However, Bandura favors a domain-specific technique for studying MPSE [7].

Nevertheless, irrespective of the apparent relationship between self-efficacy, social assistance, and postnatal stress, scant research has been conducted to investigate these interlinked elements in mothers during the early postpartum period [13]. The limited studies available are only focused on mothers in Western countries or a particular ethnic group, such as Chinese mothers residing in Hong Kong [10, 12, 15]. Despite previous research having investigated the predictors of MPSE in both primiparous and multiparous women [13], there is still a lack of understanding on the needs of women as mothers [9] and on the nature of the relationship between MPSE and other factors [7, 9]. Therefore, this study attempts to expand on previous research by investigating the predictors of MPSE among Jordanian women.

## Method

### Design

A descriptive cross-sectional approach was utilized in this research. This strategy seemed appropriate for achieving the research objectives since the aim of the study was to examine and define the association between all the factors of interest at a specific time without altering the associations between variables.

### Subjects

The study was conducted in public tertiary health facilities in Irbid City in Jordan with an annual delivery of services to 30,000 mothers. Convenience sampling was employed to select suitable participants during the hospital discharge procedure. Convenience sample was achieved by visiting tertiary health centers in Irbid city and determine mothers that met inclusion criteria and invited them to participated.This type of sampling is especially beneficial when seeking to recruit subjects within a short duration of time, which is particularly relevant for the topic under study. The inclusion criteria for selecting the women were as follows: (a) age more than 18 years old, (b) mother to a child aged less than 3 years old, and (c) ability to read and write in Arabic. The exclusion criteria were designed to omit mothers who (a) were cognitively disabled according to their medical records, and (b) had suffered extreme intra-partum, ante-partum, or postnatal maternal difficulties, since these problems may impact the mother’s self-efficacy in negative ways. This is an indication that only women without any ante-natal complications were enrolled in the study.

The estimation of the sample size relied on the use of a correlation assessment. This assessment suggested that in order to determine the association of MPSE with social assistance and other demographic factors, a minimum sample size of 200 was required. This figure was based on the need to have an effect of 0.25, and 80% power at a level of significance of .05 (two-sided). More than 250 were approached to participate in the study, but some of them did not participate for personal reasons. 220 surveys were returned, but seven were excluded since they were missing more than 20% of the data. A total of 213 women participated in the study.

### Instrument

The instrument for this study consisted of three parts: a demographic questionnaire, the State Anxiety Inventory (SAI), and the Maternal Parenting Self-Efficacy (PMP S-E) tool. The researcher developed a questionnaire to obtain background information about the sample, such as age, gender, marital status, education level of the mother, living area (urban or rural), and the number of family members. See Table 2.

### State Anxiety Inventory (SAI)

The SAI [16] was used to gauge the state of anxiety. The SAI contains concise statements in both English and Arabic. Each statement requires an answer based on a four-point scale ranging from 1 = rarely to 4 = frequently. There are 20 items in the SAI and the scores for these 20 items are summed to provide an overall score or rating. Hence the total score ranges from 20 to 80. A score of 80 shows that the respondent has high state anxiety and a result of 20 implies that the respondent has a low level of state anxiety. There is evidence that the psychometric characteristics of the SAI have good reliability as the values of the coefficients range between 0.88 and 0.92 (Cronbach’s alpha) and from 0.70 to 0.93 (test-retest). These values indicate that the SAI has excellent internal precision and consistency. In addition, the validity of the scale ranges from 0.70 to 0.88 (based on five methods, [17]). Permission to use the Arabic version of the SAI was kindly given by Dr. Abdel-Khalek.

### Maternal parenting self-efficacy (PMP S-E) tool

The PMP S-E tool [18] was used to measure the mothers’ attitudes toward self-efficacy in the care of their infant and toddlers. This tool focuses on 20 features that are grouped under four subscales: caretaking procedures, expression reading, cueing behaviors, and assumptions of a circumstance. The responses to each feature are given by the respondent based on a four-point Likert scale ranging from 1 = strongly agree to 4 = strongly disagree. Hence, the total score can vary from 20 to 80, where a greater score implies a greater degree of MPSE with a Cronbach’s alpha of 0.78 [19].

### Ethical consideration

The ethical endorsement to carry out this research was granted by the Ethical Committee of the Jordan University of Science and Technology (JUST) and the Institutional Review Board (IRB) of the Ministry of Health. The subjects were given information concerning the objectives of the study by the researcher, details of its benefits, the freedom to choose to participate, and the privilege of freely withdrawing from the survey. In addition, the subjects were given an assurance that their data would be confidential and that only the researcher could access the information.

### Data collection

Data was only collected after the IRB of the Ministry of Health and the Ethical Committed of the JUST had given approval for the study to proceed. The mothers at the facilities who agreed to participate were interviewed to determine whether they met the criteria for inclusion. If they satisfied the criteria, they were asked to sign a consent form confirming that they wished to participate before they were issued with a folder. The folder contained an introduction letter, two consent forms (one for the researcher and one for the subject), a self-sealing envelope, and a survey form. The researcher gathered data from the subjects from the facilities. Every subject at the facilities was given approximately 20 minutes to finish the survey. This was in line with a pilot study that was conducted on a sample with a diverse range of education from which it was concluded that the survey instrument would take from 15 to 20 minutes to complete. The researcher’s assistants were present during the completion of the survey in order to respond to questions, and give clarifications.

### Data analysis

The descriptive statistics were analyzed using SPSS first according to mean, frequency, and distribution. Any discrepant or unusual data was identified and dealt with before this analysis. Surveys with more than 20% of the data missing were excluded from the study. A table format was used to display the descriptive information on the attributes and socio-demographics of the participating mothers. All the statistical procedures were subjected to an assumption trial. Normality was investigated for all the continuous-level data. The assumption trial for the multiple regression analysis was done to determine the predictors of MPSE. The significance level was determined to p≥ .05. The covariates factors were mother age, mother education, mother assistance, mother employment, mother experience with children, place of living, marital relation, family income, infant age, infant gender.

## Results

### Socio-demographic variables

The age of the mothers ranged between 19 and 37, with a mean age of 30.2 years. The majority of the mothers (78%, *n* = 168) had completed a bachelor’s degree or higher. Over half were unemployed (56.3%, *n* = 120). The mean monthly income of the family was 887.2 Jordanian dinars. The age of the infant and toddlers ranged between 3 and 30 months, with a mean age of 17 months with just over half the infants and toddlers female (56.3%, *n* = 120) and the rest male (43.7%, *n* = 93). See Table 1.

**Table 1:** Characteristics of the study participants (*N = 213*).

Parameters	n (%)	Mean (SD)
**Mother age (years)**		17 (6.2)
**Educational level**		
• Primary or Secondary	45 (22)	
• Bachelor or more	168 (78)	
**Employment status**		
• Employed	94 (43.7)	
• Not employed	119 (56.3)	
**Assistance in infant care**		
• Yes	74 (34.9)	
• No	139 (65.1)	
**Assistance is provided by**		
• Child grand parents	70 (65.4)	
• Husband	30 (28.0)	
• others	7 (6.5)	
**Experience in infant care**		
• Yes	159 (74.4)	
• No	54 (25.6)	
**Living in Independent house**		
• Yes	168 (79.1)	
• No	45 (20.9)	
**Family income level (Jordanian dinar)**		887 (182.0)
**Infant age (months)**		27 (3.6)
**Planning of Pregnancy**		
• Yes	193 (69.7)	
• No	84 (30.3)	
**Mode of delivery**		
• Normal Vaginal Delivery	141 (66.4)	
• Cesarean section	72 (33.6)	
**Marital relationship**		
• Very good	97 (45.5)	
• Good	87 (40.8)	
• Fair	21 (9.7)	
• Bad	8 (3.6)	
**MPSE**		65(5.8)
**SAI**		58(4.3)

One Jordanian dinar is equal to 1.4 US dollars.

### Association between maternal parenting self-efficacy and maternal anxiety

Based on the results of the SAI, no marked relationship was found to exist between the MPSE with maternal anxiety (r = .107, P = .119). This means that maternal anxiety does not predict MPSE and does not have any impact. See Table 1 for mean score for anxiety and MPSE.

### Multiple regressions on predictors of maternal parenting self-efficacy

Multiple regression analysis revealed a significant pattern (*F* = 2.930, *P* = 0.01) that indicated that MPSE was associated with the following factors: quality of marriage relations (*B* = 3.56, *P* = 001), as a higher quality of marriage meant an increased MPSE score, family income (*B* = 1.97, *P* = .05) as the increasing of income also caused an increase in the MPSE score. Employment is another factor that impacted MPSE and showed that working mothers did not exhibit more MPSE toward infants and toddlers (*B* = -.187, *P* = .027). Education is another factor that impacted the MPSE score (*B* = .214, *P* = .004) and this means educated mothers exhibit more MPSE toward their infants and toddlers than uneducated mothers, and living with extended family (*B* = 5.28, *P* = .02) enhance the mother ability to exhibit greater MPSE. Hence, these factors were found to be key predictors of MPSE. The results of the multivariate regression analysis are shown in detail in Table 2.

**Table 2:** Multiple regressions for predictors of maternal self efficacy.

Model	Unstandardized Coefficients		Standardized Coefficients	T	Sig.
	B	Std. Error	Beta		
(Constant)	64.063	10.388		6.167	.000
Anxiety	-.130	.134	-.067	-.965	.336
Mother age	.112	.151	.055	.740	.460
Education	2.480	.854	.214	2.903	.004
Employment	-4.027	1.802	-.187	-2.236	.027
Assistance	.102	1.691	.005	.061	.952
Experience	-2.317	1.552	-.108	-1.493	.137
Living	-5.814	2.472	-.167	-2.352	.020
Marital Relation	3.568	1.021	.251	3.494	.001
Income	.001	.001	.139	1.970	.050
Infant age	.100	.071	.101	1.420	.157
Infant gender	1.396	1.444	.065	.967	.335

## Discussion

This study aims to investigate the relationship between maternal anxiety and the predictors of maternal parental self-efficacy when the children concerned are in the early years of life. Based on the outcomes of this study, it seems that environmental factors, including family factors, could have an impact on MPSE.

Similar to previous studies by Ngai et al. and Salonen et al, our study found that maternal anxiety did not predict self-efficacy. However, there is a need for a longitudinal study to gauge the association between MPSE and maternal anxiety at different times during the postnatal period [12, 14]. However, this may explain that most of Jordanian mothers experience social support from their mothers and mother-in-laws that may raise their MPSE and enhance communication and interaction with the child.

Our results showed that Jordanian mothers appreciate being in contact with their infant and toddlers, irrespective of the time taken to understand the needs of their infant and toddlers through the infant and toddlers’ expressions of temperament. In addition, Jordanian mothers with experience in caring for infants and toddlers before they had their own child and those living in a large family had an elevated level of MPSE. The above outcomes are in agreement with the results reported by Gharaibeh and Halman, who proposed that maternal background may work as precursors of self-efficacy. In the context of Arabic culture [11], these outcomes show that the practice of initiating young Arab girls into caring for infant and toddlers before marriage through participating in looking after their siblings and after infants and toddlers of members of their extended family normalizes the role of young girls in caring for infant and toddlers and prepares them for their future caring responsibilities [12]. Furthermore, the presence of social assistance for mothers in Jordan can explain the vital function of the extended family in enhancing self-efficacy. It has been shown in previous studies that the customs and traditions of Jordanians make a beneficial contribution to mothers’ perceived maternal capacity and self-efficacy in caring for and managing newborns [4, 14]. Culturally, the assistance that the mother and the mother-in-law give to first-time mothers entails educating the new mother on cleaning the baby, breastfeeding, perinatal care, and massaging the abdomen, as well as the preparation of particular foods to guarantee the sufficient production of breast milk. According to Jakalat, traditionally, progressive care takes 40 days [20].

The findings of this study also showed that mothers with experience of caring for an infant and toddler before motherhood by living with extended family, being an aunt, and participating in childcare had a lot of confidence in their ability to take on caregiving responsibilities. Those who had watched others care for infants and toddlers or had received guidance, assistance, and words of encouragement perceived that they had high levels of self-efficacy. These outcomes are consistent with the results of Farkas and Valdés and Abuhammad et al, who stressed the significance of assistance in promoting self-efficacy and courage in caring for infants and toddlers [21, 22].

According to Leahy-Warren and McCarthy [11], maternal efficacy can also be perceived as a precursor to parenting skills, and it has been shown that maternal and child attributes, interventions for antenatal care [1], and the quality of marriage [12, 23] predict MPSE. Moreover, this study’s finding that social support acts as a predictor of MPSE agrees with previous results [13–15]. These experiences are related to the culture. According to Serçekuş and Başkale [24], culture plays a significant role in the transition into motherhood. Therefore, further research centering on different ethnicities would enable researchers to gain more insight on the connection between MPSE and ethnicity.

As for the impact of age, in contrast with previous Jordanian [13] and international [15, 24] research, this study found that age of mother was not a predictor of MPSE. Previous studies showed that older mothers may have a lot of social relationships and thus have access to a lot of social assistance that can boost their MPSE. In addition, a higher family income was also found to predict greater MPSE, which is in accordance with previous research [24, 25]. Mothers in families with a higher income may have a higher likelihood of paying for a home assistant and for an in-house nanny during the postpartum period [26–28], which would consequently positively influence the MPSE of the mother.

### Nursing education and implications

It is essential for nurses to understand MPSE. Therefore, including the concept of maternal parenting self-efficacy in nursing curricula can help raise awareness of this important concept. Understanding maternal parenting self-efficacy is necessary for nurses to evaluate the mothers’ parenting self-efficacy. Certain anticipatory guidance and related interventions can be explored as required. Nurses can offer education to the mothers regarding parenting maternal self-efficacy, which will enable them to care for their children properly. For example, the nurses should teach mothers that parenting self-efficacy is modifiable. It is possible that maternal parenting self-efficacy and parenting practices may mutually influence each other over time.

### Conclusion

The findings of this study show that MPSE is significantly associated with various predictors. These predictors are the mother’s education, income, if she lives with extended family, her quality of marriage, and her employment. Maternal anxiety was found not to be a predictor for MPSE and this may explain other factors such as social support and living with extended family. Nurses may therefore wish to consider evaluating women who show a possible risk of low MPSE and assist them via training, assistance, and interventions to boost their parental self-efficacy. However, it should be noted that the theoretical technique applied in this study needs to be replicated in order to bolster these findings empirically. In addition, more studies on the impact of socio-demographic variables and other factors on MPSE are required to provide support to the outcomes of this study and deeper insight into this phenomenon.
